# The Prevalence of *Helicobacter pylori* in Estonian Bariatric Surgery Patients

**DOI:** 10.3390/ijms19020338

**Published:** 2018-01-24

**Authors:** Natalja Šebunova, Jelena Štšepetova, Toomas Sillakivi, Reet Mändar

**Affiliations:** 1 Institute of Biomedicine and Translational Medicine, University of Tartu, Ravila Street 19, 50411 Tartu, Estonia; natalja.sebunova@ut.ee (N.Š.); reet.mandar@ut.ee (R.M.); 2 Competence Centre on Health Technologies, Tiigi Street 61B, 50410 Tartu, Estonia; 3 Abdominal Surgery Department of Tartu University Hospital, Puusepa Street 8, 51014 Tartu, Estonia; toomas.sillakivi@kliinikum.ee

**Keywords:** *Helicobacter pylori*, *glmM* gene, *cagA* gene, PCR, gastritis, obesity, virulence factors

## Abstract

*Helicobacter pylori* (*Hp*) is one of the most important human pathogens that can cause duodenal and gastric ulcers, gastritis and stomach cancer. *Hp* infection is considered to be a cause of limiting access to bariatric surgery. The aim of this study was to determine the prevalence of *Hp* in patients with obesity going into bariatric surgery and to reveal the relationship between *Hp* and clinical data. The study group was formed of 68 preoperative bariatric surgery patients (body mass index (BMI) 44.7 ± 4.8). Gastric biopsies (antrum and corpus) were used for histological and molecular (*caqA* and *glmM* genes) examinations. The PCR method revealed *Hp* infection in 64.7% of obese patients that is higher in comparison with histological analysis (55.9%). The prevalence of *cagA* and *glmM* genes in antrum mucosa was 45.6% and 47.0% while in the corpus it was 41.2% and 38.3%, respectively. The coincidence of both *cagA* and *glmM* virulence genes in the antrum and corpus mucosa was 33.8% and 22.1%, respectively. Either of the genes was found in 58.8% of antrum and 57.3% of corpus mucosa. Presence of *caqA* and *glmM* genes was in association with active and atrophic chronic gastritis. In conclusion, our study demonstrated that two thirds of morbidly obese patients undergoing bariatric surgery are infected with *Hp* and have a high prevalence of *cagA* and *glmM* virulence genes that points out the necessity for diagnostics and treatment of this infection before surgery.

## 1. Introduction

*Helicobacter pylori* (*Hp*) is one of the most important human pathogens. *Hp* is a helix shaped, microaerophilic, Gram-negative bacteria. Some strains can form nonculturable coccoid form [1,2]. It colonizes and infects gastric epithelial cell surfaces and the overlying gastric mucin, which is a highly specialized niche [2]. According to the World Health Organization (WHO), more than 50% of the human population is infected [3], while over 80% of infected individuals are asymptomatic [4].

Previous studies suggest that *Hp* is genetically variable and certain genotypes may be detected only in certain populations [5,6]. Several virulence factors contribute to the inflammatory response towards *Hp* either by altering host signaling pathways important to maintaining tissue homeostasis in epithelia cells or by differentially stimulating innate immune cells [3,7]. Among the different genes involved in *Hp* pathogenicity, *glmM* (phosphoglucosamine mutase gene) and *cagA* (cytotoxin-associated gene A) virulence genes have been demonstrated to be highly sensitive predictors of several clinical outcomes [2,3,8,9,10]. In particular, the *glmM* gene is highly conserved, has a high degree of sensitivity and specificity [11], while the *caqA* gene is one of the major virulence factors in *Hp* responsible for gastric pathology [12].

Prevalence of *Hp* infection varies around the world, and previous studies on obese patients have shown conflicting results: some studies have reported a higher prevalence [13,14,15,16], while others have shown a decreased prevalence when compared to the general population [17,18,19]. *Hp* infection is considered to be limiting access to bariatric surgery [15]. To date, there are no data about its prevalence in obese preoperative bariatric surgery patients in Estonia.

The aim of this study was to determine the prevalence of *H. pylori* and its virulence genes (*cagA* and *glmM*) in the obese patients going into bariatric surgery. We also aimed to reveal the relationship between *Hp* and clinical data.

## 2. Results

### 2.1. Clinical Indices and Endoscopic Findings

The clinical indices of patients are presented in Table 1. The mean age of the patients was 45.3 ± 9.6 and the mean body mass index (BMI) was 44.7 ± 4.8. An increased white blood cell count (WBC) count was found in 22% of the subjects and raised C-reactive protein (hs-CRP) was found in 57% subjects. The level of fasting blood glucose was raised in 60% patients, while an increased low-density lipoprotein (LDL)-cholesterol level was found only in 1.5% of patients.

An endoscopic examination was performed for the esophagus and stomach, followed by a histological analysis of the biopsy samples (Table 2). The most prevalent pathology in the antrum mucosa was chronic active gastritis in 27.8% of all cases, while in the corpus mucosa the most common diagnosis was superficial chronic gastritis in 38.3% patients. In total (antrum and/or corpus mucosa), the most prevalent diagnoses were superficial chronic gastritis and active chronic gastritis in 42.7% and 32.2% of patients, respectively. No detectable histopathological changes in the stomach were found only in three patients (4.4%).

### 2.2. Prevalence of cagA and glmM Genes According to PCR Analysis

The amplification of *cagA* and *glmM* genes of *Hp* was done for all stomach biopsies (Table 3). The prevalence of *cagA* and *glmM* genes in the antrum mucosa were 45.6% and 47.0%, while in the corpus mucosa it was 41.2% and 38.3%, respectively. The coincidence of both *cagA* and *glmM* virulence genes in the antrum and corpus mucosa was 33.8% and 22.1%, respectively. In total, either of the genes was found in 58.8% and 57.3% of the antrum and corpus mucosa, respectively (Table 3).

The PCR method revealed a *Hp* infection quite similar to the histological method: 58.8% and 57.3% for the antrum and 57.3% and 55.9% for the corpus mucosa, respectively. In total, the PCR method revealed a *Hp* infection in 64.7% of obese patients that is slightly higher in comparison with histological analysis (55.9%).

### 2.3. Associations between Virulence Genes, Clinical Parameters and Histopathological Findings of Gastric Mucosa

Statistically significant correlations between the histopathological findings for the gastric mucosa and the presence of virulence genes are presented in Table 4. The presence of precancerous conditions (atrophic gastritis and/or metaplasia) in the antrum mucosa was associated with the presence of *cagA* and *glmM* genes in 50.0% for both genes of all cases, while for the corpus mucosa this was 50.0% and 29.4%, respectively (Table 5). The prevalence of the *cagA* gene was lower in patients without atrophic gastritis and metaplasia, however, this difference was not statistically significant.

There were no correlations between patients’ gender, age, BMI and the presence of *Hp* in the antral and corpuscular gastric mucosa. No statistical differences in blood parameters were found between *Hp* positive and negative patients, though, hs-CRP was increased in 63.6% (28/44) of *Hp* positive obese patients (Table 6).

## 3. Discussion

The present study revealed a remarkably high prevalence of both gastritis (96%) and *Hp* infection (65%) among preoperative bariatric surgery patients, the latter being confirmed by two methods, PCR and histological analysis. *Hp* virulence genes, *caqA* and *glmM* were associated with different forms of chronic gastritis both in the antrum and corpus. *Hp* was not associated with the demographic data and blood parameters of obese patients.

According to the literature, mostly serology, stool antigen assays and urea breath tests have been used for the detection of *Hp* infection in obese patients [15]. Histological investigation also has excellent sensitivity and specificity, especially when specific immunostaining is used [15,20]. Vanek et al. have compared serology and histology and found good accordance between the two methods [21]. In our study, we also combined two approaches: histology and the detection of *H. pylori* virulence genes such as *glmM* and *cagA* by the polymerase chain reaction (PCR) method.

Among the different genes involved in the pathogenicity of *Hp,* the *glmM* (phosphoglucosamine mutase gene) and *cagA* (cytotoxin-associated gene A) virulence genes have been demonstrated to be highly sensitive predictors of severe clinical outcomes [2,3,9]. *CagA* is the most-studied virulence factor of *Hp*, which is a 120–145 kDa protein encoded on the *cag* pathogenicity island (PAI) [22]. It contains 31 potential coding regions which encodes a type IV secretion system (T4SS) through which *cagA* is delivered into host cells [23,24,25]. Phosphorylated or nonphosphorylated *cagA* can interact with host proteins that regulate cell growth, cell motility and cell polarity altering host cell signaling. Thus, *cagA* can promote cells to accumulate multiple genetic and epigenetic changes involved in gastric carcinogenesis and gastric adenocarcinoma development [26]. Also, it is highly antigenic, inducing interleukin-8 (IL-8) secretion by gastric epithelial cells [27]. It is one of the mechanisms involved in forming the neutrophilic infiltration of epithelium and mucous. Such pathological processes can lead to the formation of lymphoid follicles and epithelial damage of varying severity [8,28].

The housekeeping *glmM* gene encodes a phosphoglucosamine mutase, an enzyme catalyzing the interconversion of glucosamine-6-phosphate into glucosamine-1-phosphate, which is subsequently transformed into *N*-acetylglucosamine. This monosaccharide is one of the main cytoplasmic precursors of bacterial cell wall murein and outer membrane lipopolysaccharides. Consequently, the *glmM* gene is essential for bacterial cell growth and assists directly with cell wall synthesis. The *glmM* gene is highly conserved between strains [29,30]. The presence of the *glmM* gene in *Hp*-positive obese persons has not been described before. One of the advantages of using this gene to identify *Hp* is its high sensitivity and specificity, since it has the detection rate of 10 to 100 *H. pylori* cells, which is significantly better than histology [31].

Previous studies have shown that the *Hp*-containing *cagA* gene is associated with the development of chronic active gastritis (AG) [32], peptic ulceration [8,10,33] and athrophic gastritis with an increased risk of gastric cancer [34,35] while the *glmM* gene is associated with the development of chronic superficial gastritis as well as intestinal metaplasia (IM), gastric ulcers and gastric dysplasia, and it was less expressed in chronic gastric ulcers and atrophic gastritis [36,37]. Our study revealed that both genes were associated with chronic active and atrophic gastritis in the antrum and corpus, while the presence of the *glmM* gene was associated with chronic active gastritis in the antrum, which is different from the data of Helaly et al. [36]. Also, a half of the morbidly obese patients suffered from atrophic gastritis and/or metaplasia in our study, which are the main precursors of gastric cancer. According to Correa’s theory, the formation of gastric cancer is a multistep and multifactorial process where the presence of *Hp* infection is one of the main factors. Gastritis begins from superficial gastritis and may progress into atrophic gastritis, metaplasia, dysplasia and gastric cancer [36]. The majority of studies determining the prevalence of AG and IM around the world touch on the general population, but not morbidly obese patients. The prevalence of AG and IM in Estonian obese patients is quite similar to the data of Korean, Chinese and Japanese rural populations (42.7%, 63.8% and 55.5% for AG, respectively) [37,38,39] while it differs from studies of German (6.0% for AG), Swedish (0.6% for AG) and US rural populations (15.0% for IM) [40,41,42].

Using a combination of two tests, our study determined the prevalence of *Hp* in preoperative bariatric patients in Estonia to be 64.7%. To our knowledge, this report is the first to reveal the virulence factors of *Hp* among preoperative bariatric patients in Estonia, although *Hp* in Estonian obese patients has been detected earlier using the serological method, with a prevalence of 51.7% [38]. Our study did not include a non-obese control group, but previous studies have revealed the *Hp* prevalence in the adult Estonian population to be 69% [43], which is nearly similar to the present study. *Hp* infection has large disparity between developed and developing countries [39]. Although Estonia belongs to the European countries, the prevalence of *Hp* infection is still quite high in comparison to other countries.

According to the previous data, the prevalence of *Hp* infection in morbidly obese patients is still controversial. It has been shown to vary from 2.2% in Australia [42] and 8.7% in Germany [40] to 85.5% in a Saudi cohort [41]. The increased prevalence (40.93%) of *Hp* infection was found in Chinese patients with higher BMI levels in comparison with lower ones [44]. At the same time, some studies have shown no correlation [45,46] or a negative correlation [42] between obesity and the prevalence of *Hp* infection. A study of the Japanese population has shown lower BMIs in patients with gastritis than in patients without gastritis [47]. In our study we did not find an association between *Hp* and BMI. The question why infected and not infected morbidly obese patients may have similar BMIs, remains to be answered. The knowledge of the pathophysiology between *Hp* and obesity is limited due to the complex nature of the organism [46]. It is possible that many factors such as geographical region and social status can play a role in the pathogenesis of *Hp* infection in certain obese patients. A study by Fontana et al. [48] demonstrated that agonist-stimulated production of interferon-γ (IFN-gamma) and macrophage chemoattractant protein-1 (MCP-1) are significantly suppressed in subjects with obesity. Weight loss completely normalizes the ability of stimulated peripheral blood mononuclear cells (PBMCs) to produce MCP-1 and IFN-gamma. Thus, obese patients have an increased risk of bacterial and viral infections [48].

Our study did not reveal an association between *Hp* and age that is in accordance with that of Helaly et al. [34]. However, contrary data have been shown by some other studies [45,46] where obese patients with *Hp* infection were significantly older compared to those without infection. This tendency can be related to alterations in immune system functions during human organism ageing as well as lower hygiene levels during the youth of elderly people.

We found that more than half of *Hp* positive obese patients had increased hs-CRP levels in serum, which is in accordance with other data [49]. In a study of Turkish patients it was demonstrated that serum levels of hs-CRP were significantly reduced in most *Hp* positive patients after antibiotic eradication therapy [50].

A previous animal study has demonstrated that *Hp* colonization may decrease fasting blood glucose levels and improve glucose tolerance [51]. In our study, mean glucose levels tended to be higher in *Hp* positive patients, but due to the small sample size this tendency was not statistically significant.

There were a few limitations in our study. Firstly, we did not have a control group for comparison of the prevalence of *Hp* among the healthy population that may have considerably changed in recent years. Secondly, the current study was limited because of the small sample size (*n* = 68).

## 4. Materials and Methods

### 4.1. Patients

The study was carried out at Department of Microbiology, Institute of Biomedicine and Translational Medicine, University of Tartu in collaboration with Tartu University Hospital. The study group was formed of 68 patients (46 females, 22 males; age range: 19–65 years, mean age 45.3 ± 9.6 years; mean body mass index (BMI) 44.7 ± 4.8) attending the Surgery Clinic of Tartu University Hospital between March 2015 and December 2016. BMI was calculated as described [52]. Blood samples were obtained after 8 h of fasting and stored immediately at 4 °C. Laboratory analyses were performed with standard methods using certified assays in the United Laboratory of Tartu University Hospital. Intervals for routine laboratory tests proposed by the Nordic Reference Interval Project (NORIP, available online: http://www.furst.no/norip/) were used as references.

### 4.2. Ethics Statement

The present study was conducted according to the guidelines laid down in the Declaration of Helsinki. The study was approved by the Ethics Review Committee on Human Research of Tartu University, Estonia (protocol No. 244/T-15, 17 February 2015 of approval). Participation in the study was voluntary. Written informed consent was obtained from all study subjects.

### 4.3. Endoscopic Examination of the Esophagus, Stomach and Duodenum

Gastrointestinal endoscopy was performed by an experienced endoscopist on an empty stomach (no taking food at least four hours before procedure). In every patient, two biopsies from the gastric antrum (2 cm from the pyloric ring) and two biopsies from the corpus were collected for histological evaluation; the same package of biopsies was collected for molecular studies. The stomach and esophagus were examined; and all the endoscopic findings were registered.

### 4.4. Gastric Biopsies Processing

Formalin-fixed, paraffin-embedded gastric biopsy specimens from the antrum and corpus mucosa were stained with hematoxylin and eosin, and with a modified Giemsa stain. The state of the gastric mucosa and the presence of *Hp* in histological sections were assessed to grade the severity of gastritis, intestinal metaplasia and presence of *Hp* infection. The remaining biopsies used for molecular applications were stored frozen at −80 °C until processed.

### 4.5. Molecular Analysis

The frozen biopsy specimens were suspended in 500 µL of lysis buffer (200 mM Tris-HCl (pH 8.0), 25 mM ethylenediaminetetraacetic acid (EDTA), 300 mM NaCl, 1.2% sodium dodecyl sulfate) and 20 µL of proteinase K (400 µg/mL) for DNA extraction. The mixture was incubated at 37 °C for 24 h. The procedure of DNA extraction was continued according to the tissue protocol of QIAamp DNA Blood Mini Kit (Qiagen, Hilden, Germany). Extracted DNA was used for subsequent PCR experiments. The DNA amplification was performed in reaction volume of 50 µL containing 25 µL of Hot Start Buffer (Fermentas, Vilnius, Lithuania); 1 µM of each primer, (5′-ATA ATG CTA AAT TAG-3 and 5′-TTA GAA TAA TCA AGA-3′ for amplification the *cagA* gene; 5′-AAG CTT TTA GGG GTG-3′ and 5′-AAG CTT ACT TTC TAA-3′ for amplification *glmM* gene and 5 µL of extracted DNA [9,53,54]. The mixtures were placed into a PCR thermocycler (Mastercycler gradient, Eppendorf, Hamburg, Germany). PCR conditions were as follows: an initial denaturation (94 °C, 2 min), 35 cycles of denaturation (94 °C, 2 min), annealing (55 °C, 2 min), and extension (72 °C, 2 min), with a final extension (72 °C, 10 min). PCR products were identified using agarose gel electrophoresis on 2% agarose gel, ethidium bromide staining, and UV transillumination.

### 4.6. Statistical Analysis

For statistical analysis, SigmaPlot 12.0 (Systat Software Inc., San Jose, CA, USA) software was used. The differences between the groups were analyzed using the Mann–Whitney rank sum test and *t*-test. Spearman rank order correlation analysis was used to find associations between the markers. Statistical significance was assumed at *p* < 0.05 for all parameters.

## 5. Conclusions

In conclusion, our study demonstrated that two thirds of morbidly obese patients undergoing bariatric surgery in Estonia are infected with *Hp*, while the prevalence of the virulence genes *cagA* and/or *glmM* accounted for nearly 60% of cases. The presence of these genes is associated with a high prevalence of active and atrophic chronic gastritis that in turn bears higher risks of serious consequences for the patients. The pathological changes and processes induced by *Hp* in the gastric mucosa may interfere with the welfare and health of operated subjects in long run, which points out the necessity for the diagnosis and treatment of this infection before surgery.

## Figures and Tables

**Table 1:** Clinical data of study subjects (mean ± SD, range).

**Anthropometric Parameters**				
**Parameter**		**Male (*n* = 22)**	**Female (*n* = 46)**	**Total (*n* = 68)**
Age (years)		45.6 ± 10.1 (29–65)	45.2 ± 9.5 (19–61)	45.3 ± 9.6 (19–65)
Height (cm)		180.6 ± 8.2 (160.0–198.0)	165.4 ± 6.3 (150.0–180.0)	170.4 ± 10.0 (150.0–198.0)
Weight (kg)		158.9 ± 34.6 (105.0–240.0)	119.4 ± 14.0 (95.0–167.0)	131.5 ± 28.7 (95.0–240.0)
BMI (kg/m^2^)		44.8 ± 5.2 (37.0–53.0)	44.6 ± 4.6 (35.0–53.0)	44.7 ± 4.8 (35.0–53.0)
**Blood Parameters**				
**Blood Parameter**		**Reference Range**	**Mean ± SD (Range)**	
WBC (×10^9^/L)		3.5–8.8	7.8 ± 2.7 (3.5–17.0)	
hs-CRP (mg/L)		<5	10.3 ± 11.1 (1.0–62.0)	
Hgb (g/L)	male	135–160	150.7 ± 14.1 (121.0–182.0)	
	female	120–140	135.3 ± 14.1 (81.0–161.0)	
Glycose (mmol/L)		3.0–5.6	6.5 ± 2.2 (4.3–14.4)	
Cholesterol (mmol/L)		3.9–7.8	5.0 ± 1.1 (2.9–7.8)	
LDL-cholesterol (mmol/L)		2.0–5.3	3.2 ± 1.1 (1.1–6.2)	

Legend: BMI-body mass index; WBC-white blood cell count; Hgb-hemoglobin; hs-CRP-C-reactive protein; LDL-low-density lipoprotein.

**Table 2:** Endoscopic findings in patients with obesity (number and %).

Endoscopic Findings		Antrum Mucosa Only	Corpus Mucosa Only	Both Antrum and Corpus	Antrum and/or Corpus Mucosa
Chronic gastritis	active	13 (19.0)	3 (4.4)	6 (8.8)	22 (32.2)
	atrophic	12 (17.7)	3 (4.4)	2 (2.9)	17 (25.0)
	active and atrophic	9 (13.2)	2 (2.9)	2 (2.9)	13 (19.0)
	active and atrophic; with metaplasia	3 (4.4)	0 (0)	0 (0)	3 (4.4)
	atrophic; with metaplasia	2 (2.9)	2 (2.9)	0 (0)	4 (5.8)
	type unspecified	9 (13.2)	4 (5.9)	2 (2.9)	15 (22)
	type unspecified; with metaplasia	0 (0)	1 (1.5)	0 (0)	1 (1.5)
	superficial	3 (4.4)	25 (36.8)	1 (1.5)	29 (42.7)
	superficial; with metaplasia	1 (1.5)	0 (0)	0 (0)	1 (1.5)
No pathology		-	-	3 (4.4)	-

**Table 3:** Prevalence of virulence genes and *H. pylori* infection according to PCR and histological analysis in obese patients (number and %).

Biopsy Sample	Method of Analysis/Gene	Positive
Antrum	Histological analysis	39 (57.3)
	Only *cagA*	8 (11.8)
	Only *glmM*	9 (13.2)
	Both *cagA* and *glmM*	23 (33.8)
	*cagA* and/or *glmM*	40 (58.8)
Corpus	Histological analysis	38 (55.9)
	Only *cagA*	13 (19.1)
	Only *glmM*	11 (16.2)
	Both *cagA* and *glmM*	15 (22.1)
	*cagA* and/or *glmM*	39 (57.3)
Total	Histological analysis	38 (55.9)
	PCR analysis	44 (64.7)

**Table 4:** Correlations between histological findings and present virulence genes.

Histological Findings	Virulence Genes	Correlation Coefficient (r)	*p*-Value
Chronic active gastritis (antrum mucosa)	*glmM* (antrum mucosa)	0.27	0.028
	*glmM* (corpus mucosa)	0.32	0.008
Chronic active gastritis (corpus mucosa)	*glmM* (antrum mucosa)	0.33	0.007
	*cagA* (antrum mucosa)	0.25	0.038
Chrnoic active and atrophic gastritis (corpus mucosa)	*cagA* (antrum mucosa)	0.27	0.024
	*cagA* (corpus mucosa)	0.30	0.014

**Table 5:** Association between patients with atrophic gastritis and/or metaplasia and *H. pylori* (number and %).

Diagnosis	Antrum		Corpus	
	*CagA* Gene	*GlmM* Gene	*CagA* Gene	*GlmM* Gene
Patients with atrophic gastritis and/or metaplasia (*n* = 34)	17 (50.0)	17 (50.0)	17 (50.0)	10 (29.4)
Patients without atrophic gastritis and/or metaplasia (*n* = 34)	14 (41.2)	15 (44.1)	11 (32.4)	16 (47.1)

**Table 6:** Blood parameters of patients with and without *H. pylori* infection (mean ± SD, range).

Blood Parameter		Both Antrum and Corpus		*p*-Value *
		*H. pylori* Positive	*H. pylori* Negative	
WBC (×10^9^/L)		7.4 ± 2.1	7.9 ± 3.2	0.842
hs-CRP (mg/L)		10.2 ± 10.9	7.3 ± 7.2	0.205
Hgb (g/L)	male	150.0 ± 14.6	152.4 ± 12.1	0.669
	female	135.6 ± 11.5	134.7 ± 18.1	0.973
Glycose (mmol/L)		6.7 ± 2.0	6.0 ± 1.8	0.154
Cholesterol (mmol/L)		5.3 ± 1.1	5.0 ± 0.9	0.238
LDL-cholesterol (mmol/L)		3.4 ± 1.2	3.1 ± 0.9	0.369

* Mann–Whitney rank sum test was used for comparison of the groups, except for the male hemoglobin (Hgb) where *t*-test was used due to parametric distribution of data.
